# Sample preparation of *Hedyotis diffusa* Willd. for two-dimensional gas chromatography-time of flight mass spectroscopic analysis enhanced chemical profiling

**DOI:** 10.1016/j.pscia.2024.100056

**Published:** 2024-11-10

**Authors:** Duxin Li, Xinying Du, Wanru Bai, Oliver J. Schmitz

**Affiliations:** a College of Pharmaceutical Science, Soochow University, Ren’Ai Road 199, 215123, Suzhou, PR China; b Applied Analytical Chemistry, Faculty of Chemistry, University of Duisburg-Essen, Universitaetsstr. 5-7, 45141, Essen, Germany

**Keywords:** Chemical profiling, *Hedyotis diffusa* willd, Two-dimensional gas chromatography-time-of-flight mass spectrometry, Sample preparation, Herbal medicine

## Abstract

*Hedyotis diffusa* Willd., a herbal remedy for cancer, has a complex chemical profile that is difficult to analyze in detail. Here, we introduce an optimized sample preparation method coupled with two-dimensional gas chromatography-time-of-flight mass spectrometry (GC ​× ​GC-TOF/MS) to enhance the chemical profiling of *H. diffusa*. By employing dichloromethane extraction for nonpolar compounds, aqueous extraction, and solid-phase extraction fractionation into moderately polar and polar fractions, we extracted and analyzed a comprehensive range of chemicals from *H. diffusa*. GC ​× ​GC-TOF/MS analysis revealed a rich chemical landscape, identifying 185, 325, and 483 peaks in the dichloromethane extract, solid-phase extraction elution, and unretained fractions, respectively. Library matching against known compounds confirmed 155, 178, and 184 hits with a similarity of 80% or greater. Notably, this method also involves group-type elution of steroids and anthraquinones, facilitating the identification and screening of similar compounds. This comprehensive approach to herbal chemical analysis offers a high-dimensional perspective and greatly advances our understanding of the chemical constituents of *H. diffusa*.

## Highlights

• Combinational sample preparation acquired comprehensive chemical constitutes.
• GC ​× ​GC-TOF/MS analysis enhanced chemical profiling of *Hedyotis diffusa.*
• Group-type separation facilitied identification of novel steroid and anthraquinone.

## Graphical abstract

Sample preparation of *Hedyotis diffusa* Willd. for two-dimensional gas chromatography-time of flight mass chromatography (GC ​× ​GC-TOF/MS) analysis enhanced chemical profiling, including dichloromethane extraction (DCM Extract), aqueous extraction and solid phase fractionated into unretained (SPE Unretained) and elution (SPE Elution) for nonpolar, polar and moderately polar compounds, respectively.

**Figure:**
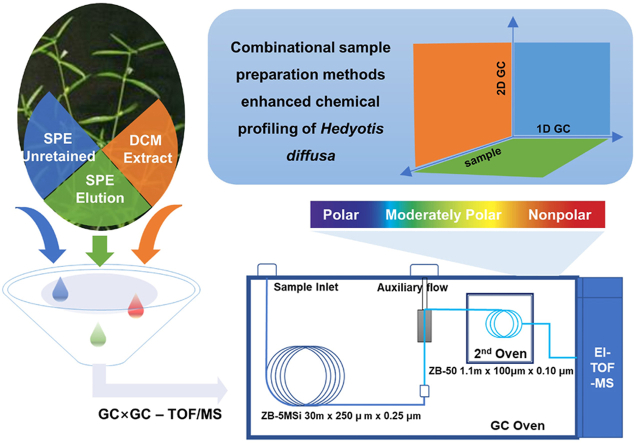

## 1 Introduction

In recent decades, the isolation and identification of compounds from plants has resulted in the discovery of bioactive chemicals with pharmaceutical, functional, and cosmetic applications [1]. However, discovering novel chemicals remains extremely difficult because most high-content compounds have already been reported. Researchers must typically start by extracting several kilograms to explore novel compounds [2] present at minor levels. Preliminary knowledge of the chemical composition of a plant before initiating the isolation process can facilitate this process.

Chromatographic-mass spectrometry (MS)-based chemical profiling is a powerful tool for determining the chemical composition of plants. One-dimensional chromatography (gas chromatography (GC)-MS [3,4] and liquid chromatography (LC)-MS [5,6]) and two-dimensional LC (2D-LC) [[7], [8], [9], [10]] have been explored for this purpose. Two-dimensional chromatographic methods have advantages over one-dimensional chromatographic methods such as a higher peak capacity, which provides greater resolving power. We previously investigated 2D-LC coupled with quadrupole time-of-flight mass spectrometry (2D‒LC QTOF/MS) [11] and detected more than 200 peaks in *Hedyotis diffusa* Willd. Moreover, we explored higher-dimensional separation via additional dimensions of ion mobility spectrometry to increase separation capability [12,13]. However, the lack of a generally accepted chemical database for LC-MS makes identifying these peaks extremely difficult.

Databases containing information on GC‒MS or 2D GC (GC ​× ​GC) coupled with MS are widely available for chemical matching. Thus, unlike LC-MS, GC-MS can be used for chemical profiling. The sample preparation method is critical for acquiring as many chemicals as possible before GC-MS analysis. Hydrodistillation [14,15] and solvent (hexane [16], dichloromethane [17], among others) extraction have been utilized for chemical profiling of various plants, particularly their essential oils. Recently, headspace [18] and solid-phase microextraction techniques [19] were developed as sample preparation methods for GC-MS. However, the complex chemical compositions of plants remain a challenge for natural products studies. Comprehensive extraction methods are needed to acquire as many chemicals as possible, and a decreased sample complexity is needed to decrease the burden of chromatographic separation, which shows co-elution peaks. Thus, a comprehensive sample preparation method combined with the separation capability of GC ​× ​GC can be used to enhance the chemical profiling of plants.

*Hedyotis diffusa*, which belongs to the *Rubiaceae* family, is widely used to treat tonsillitis, appendicitis, dysentery, and especially cancer [[20], [21], [22]]. This plant contains iridoid glycosides, flavonoids, triterpenoids, anthraquinones, sterols, and phenylpropanoids [5,23] and was used in the present study to develop a combined sample preparation method. *Hedyotis diffusa* was prepared via dichloromethane (DCM), aqueous extraction, and solid-phase extraction (SPE) fractionation. Combined sample preparation coupled with GC ​× ​GC-MS analysis was used for the chemical profiling of *H. diffusa*.

## 2 Materials and methods

### 2.1 Materials

N-Methyl-N-trimethylsilyltrifluoroacetamide (MSTFA), N,O-*bis*-tri-methylsilyltrifluoroacetamide (BSTFA), and ChromBond C18ec SPE (3 ​mL) were purchased from Macherey–Nagel (Düren, Germany). LC-MS-grade DCM and acetonitrile (ACN) were purchased from VWR International (Germany). Dried whole *H. diffusa* plants were purchased from a local apothecary in Wuppertal, Germany.

### 2.2 Instruments

Comprehensive GC ​× ​GC was performed using a 6890N gas chromatograph (Agilent Technologies, Santa Clara, CA, USA). The GC was attached to a time-of-flight mass spectrometer (TOF-MS, Pegasus III) from the LECO Corporation (St. Joseph, MI, USA). Chromatograms and MS data were stored and processed with ChromaTOF™ software (version 3.22) from LECO Corporation. A four-jet modulator operating with liquid nitrogen (cold jets) and gaseous nitrogen (hot jets) was used as the cryointerface with a modulation time of 4 ​s. The column used in the first dimension was a ZB-5MSi 30 ​m ​× ​250 ​μm ​× ​0.25 ​μm; and in second dimension was a ZB-50 1.1 ​m ​× ​100 ​μm ​× ​0.10 ​μm. The carrier gas was helium, which was applied at 1.4 ​mL/min in split-less inlet mode. The front inlet temperature was set at 310°C, and the transfer line temperature was set at 300°C. The temperature program ranged from 40°C (held for 1 ​min) to 300°C (duration of 5 ​min) at 4°C/min, and to 320°C (duration of 10 ​min) at 10°C/min. We used a second oven to independently control the temperature of the second-dimensional column. The temperature was 20°C higher than that of the first-dimension. The modulation time in the second dimension was 4 ​s. The MS acquisition delay was 500 ​s, mass range was 50–600 ​Da, detector voltage was 1600 ​V, electron energy (V) was −70e, and ion source temperature was 200°C. The MS data collection rate was 100 ​Hz.

### 2.3 Sample preparation

One gram of *H. diffusa* was immersed in 40 ​mL of water for 30 ​min and boiled for 1 ​h. The supernatant was collected, and the residual herb was boiled with an additional 30 ​mL of water for another 30 ​min. The supernatants were collected and combined as the aqueous extracts.

The SPE cartridge was activated with 3 ​mL of ACN and equilibrated with 3 ​mL of H_2_O before use. The aqueous extract (3 ​mL) was loaded into the cartridge. The flow-through solvent and eluent, obtained by rinsing with 3 ​mL 5% ACN, were collected to produce an unretained fraction. The unretained fraction was freeze-dried (2.4 ​mg); 20 ​μL of pyridine, and 450 ​μL of silylation mixture (BSTFA: MSTFA ​= ​5:1) were added; and the mixture was allowed to react at 80°C for 60 ​min before analysis. This sample was labeled as SPE Unretained.

The cartridge was evacuated to dry, and 3 ​mL of ACN was applied to elute the residues of the aqueous extract. The eluent was collected and concentrated to approximately 200 ​μL and labeled as the SPE Elution fraction.

DCM extraction was performed by soaking 0.30 ​mg ​*H. diffusa* powder in 3 ​mL of CH_2_Cl_2_ and extracting it twice under ultrasonication for 20 ​min. The solution was filtered through a 0.20 ​μm filter and labeled the DCM extract.

## 3 Results and discussion

### 3.1 Development of combinational sample preparation method

Researchers examining essential oils have extensively used hydrodistillation extraction for sample preparation. The chemicals extracted using this method include terpenoids, aromatic compounds, and aliphatic compounds [24], which are mostly non-polar. Solvent extraction using n-hexane or petroleum ether can yield similar chemicals. Various solvents are used to extract chemicals from plants. The polarity indices for hexane, petroleum ether, ethyl ether, DCM, and ethyl acetate were 0.1, 0.1, 2.8, 3.1, and 4.4, respectively. DCM is a dipolar solvent (dipolar moment: 1.14 D) and good solvent for hydrophobic molecules (log K ​= ​1.25). DCM was used to extract moderately polar and non-polar compounds from *H. diffusa*.

**Fig. 1:**
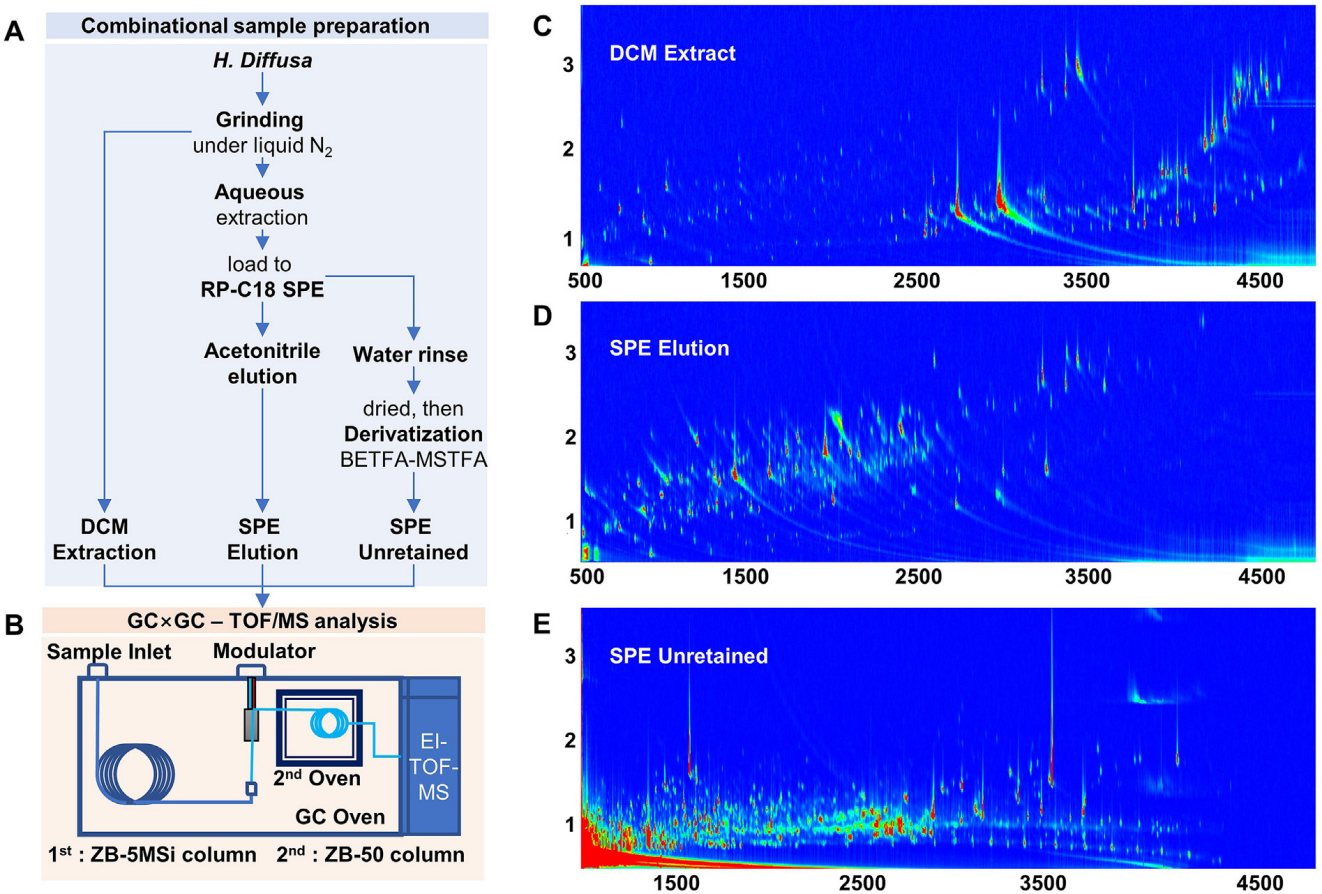
Procedure and results of comprehensive sample preparation method in combination with two-dimensional gas chromatography-time-of-flight mass spectrometry (GC ​× ​GC–TOF/MS) analysis A. Sample preparation procedure. B. Setup of the GC ​× ​GC–TOF/MS system. C. Total ion current (TIC) of GC ​× ​GC analysis of DCM Extract. D. TIC of GC ​× ​GC analysis of solid-phase extraction (SPE) Elution. E. TIC of GC ​× ​GC analysis of SPE Unretained.

Aqueous extraction is a widely used sample preparation technique for high-performance LC (HPLC) analysis. To render the extract suitable for GC analysis, fractionation via SPE was performed to separate it into moderately polar (SPE Elution) and polar (SPE Unretained) compounds. The unretained compounds in the C18 SPE cartridge contained a large amount of water and likely exhibited low volatility. Therefore, this fraction was freeze-dried and derivatized via silylation before GC analysis. The sample preparation procedure is illustrated in Fig. 1A. The combination of non-polar (DCM), moderately polar (SPE Elution), and polar (SPE Unretained) compounds provided comprehensive information on *H*. *diffusa*.

### 3.2 Optimization of GC ​× ​GC analysis conditions

GC ​× ​GC-TOF/MS can be used to profile complex samples with a high peak capacity. A critical factor affecting GC ​× ​GC performance is the orthogonality of the 2D separation columns. Typically, the columns used are combinations of nonpolar and moderately polar stationary phases: either as nonpolar ​× ​moderately polar, or moderately polar ​× ​nonpolar. A combination of a nonpolar column in the first dimension (^1^D) and a moderately polar column in the second dimension (^2^D) was used in our previous study [25]. This combination was tested via separating the DCM extract of *H. diffusa* using ZB-5MSi (5% phenyl) in ^1^D and ZB-35HT (35% phenyl) in ^2^D. However, peak tailing and wrap-round problems were observed. The separation was optimized by changing the following conditions: 1) The temperature of the ^2^D oven was increased from 10°C to 20°C higher than that of the ^1^D oven. The GC ​× ​GC system utilized a dual-oven setup. The secondary column was installed in a secondary oven to provide flexibility in optimizing secondary retention. (2) The film thickness of the ^2^D column was decreased from 0.18 to 0.10 ​μm. 3) The ^2^D column was replaced with a ZB-50 (50% phenyl) column to increase the orthogonality of the column combination.

The results of the GC ​× ​GC separation under optimized conditions are shown in Fig. 1. Separation of the DCM Extract showed large coverage of the 2D separation space, suggesting good separation. Thus, the same GC ​× ​GC conditions were used to separate the SPE Elution and SPE Unretained samples. Chromatographers seek good separation in 2D chromatography by increasing the orthogonality of either GC ​× ​GC or LC ​× ​LC analysis. The spatial coverage of the contour plot is an index of orthogonality [24]. However, orthogonality relies on both the column combination and chemical diversity of the sample. Although the large spatial coverage in the contour plots indicated good orthogonality of the column combinations in GC ​× ​GC, this coverage in contour plots requires greater diversity in the chemical composition of the sample. Thus, the sample should contain diverse chemicals, which is an important requirement for sample preparation methods.

The DCM and SPE elution samples cover a large contour plot space, suggesting that diverse chemicals were extracted. Moreover, the three extracts did not cover the same separation space, suggesting that they have different chemical compositions. Thus, different sample preparation methods produced different compounds, which is beneficial for chemical profiling. We extracted diverse compounds and provided extensive information for profiling chemicals in the herb using a combination of different sample preparation methods. Furthermore, the combination of different sample preparation methods decreased the complexity of the sample and degree of coelution to some extent. However, the unretained SPE was not well separated. Therefore, further optimization of the GC ​× ​GC conditions is required.

### 3.3 Library matching

The GC-MS database provided a priori compounds for library matching. Tentative identification of the chemicals present in *H. diffusa* was performed by searching the Wiley Registry® of Mass Spectral Data, as shown in Table 1, Table 2, Table 3, for the DCM Extract, SPE Elution and SPE Unretained samples, respectively.

**Table 1:** Compounds tentatively identified in the dichloromethane (DCM) Extract.

No.	Name	Similarity %	t_1D_ (s)	t_2D_ (s)	Area%
1	5,9-Dodecadien-2-one, 6,10-dimethyl-, (E,E))-	94	612	1.575	0.49
2	o-Xylene	95.3	640	1.035	0.43
3	Pentanoic acid	96.3	656	1.155	0.22
4	Ethane, 1,1,2,2-tetrachloro-	88.3	728	1.355	1.63
5	Methanesulfonylacetic acid	96.1	740	2.275	0.90
6	Benzaldehyde	90.2	836	1.555	0.15
7	Hexanoic acid	95.6	868	1.28	1.85
8	Furan, 2-pentyl-	91.4	900	1.105	0.67
9	Cyclobutane, 1,2-bis(1-methylethenyl)-, trans-	89	988	1.095	0.25
10	Benzyl Alcohol	91.1	1000	1.585	0.94
11	Octanoic Acid	93.7	1292	1.265	0.14
12	N(N′-Methyl-N′-nitroso(aminomethyl))benzamide	94.8	1292	1.6	0.12
13	Borneol	93.6	1296	1.34	0.32
14	Benzothiazole	95.5	1424	1.9	0.13
15	Cyclohexane, isothiocyanato-	87.3	1440	1.605	0.13
16	Nonanoic acid	90	1496	1.265	0.14
17	2(4H)-Benzofuranone, 5,6,7,7a-tetrahydro-4,4,7a-trimethyl-	89.5	2024	1.97	0.14
18	Hexadecanal	95.2	2316	1.25	0.21
19	1-Undecene, 5-methyl-	84.9	2344	1.035	0.15
20	Tetradecanoic acid	88.4	2388	1.32	0.44
21	3,7,11,15-Tetramethyl-2-hexadecen-1-ol	88.1	2512	1.105	1.51
22	2-Pentadecanone, 6,10,14-trimethyl-	88.5	2520	1.225	1.06
23	Pentadecanoic acid	90.4	2544	1.345	0.82
24	1,2-Benzenedicarboxylic acid, butyl 2-methylpropyl ester	89.2	2560	1.67	1.52
25	Hexadecanal, 2-methyl-	83	2628	1.17	0.11
26	Hexadecenoic acid, Z-11-	91	2664	1.38	0.59
27	n-Hexadecanoic acid	90.7	2704	1.435	19.88
28	cis-13-Eicosenoic acid	83.2	2808	1.395	0.24
29	Tridecanoic acid	88.2	2836	1.33	0.44
30	5-Eicosene, (E)-	88.7	2860	1.27	0.13
31	Phytol	87.3	2904	1.26	0.45
32	9,10-Anthracenedione, 2-methyl-	89.6	2912	2.405	0.15
33	9-Octadecynoic acid	84	2940	1.505	19.50
34	Octadecanoic acid	84	2976	1.36	2.08
35	1-Iodo-2-methylundecane	91.7	3136	1.115	0.29
36	2-Propenoic acid, oxybis(methyl-2,1-ethanediyl) ester	87	3136	1.28	0.12
37	Danthron methyl derivative	81.6	3196	2.75	0.92
38	4,8,12,16-Tetramethylheptadecan-4-olide	91.6	3212	1.465	1.16
39	Eicosanoic acid	82.6	3220	1.345	0.20
40	2-Tridecen-1-ol, (E)-	90.5	3296	1.305	0.16
41	2-(Hydroxymethyl)anthraquinone	81.9	3336	3.04	0.36
42	Octadecanal	93.1	3412	1.315	0.13
43	Z-2-Octadecen-1-ol	90.2	3524	1.32	0.26
44	2-Nonadecanone	92	3608	1.33	0.27
45	2,6,10,14,18,22-Tetracosahexaene, 2,6,10,15,19,23-hexamethyl-, (all-E)-	93.9	3728	1.41	10.04
46	(E,E,E)-3,7,11,15-Tetramethylhexadeca-1,3,6,10,14-pentaene	86.1	3752	1.455	0.17
47	2-Nonadecanone	92.4	3816	1.355	0.25
48	2,6,10-Dodecatrien-1-ol, 3,7,11-trimethyl-, (E,E)-	88	3856	1.515	0.85
49	1,6,10,14-Hexadecatetraen-3-ol, 3,7,11,15-tetramethyl-, (E,E)-	83.8	3900	1.555	0.19
50	Cholesta-4,6-dien-3-ol, (3βá)-	85.1	3900	1.755	0.21
51	Geranylgeraniol	84.4	3916	1.57	0.09
52	Cholesta-6,22,24-triene, 4,4-dimethyl-	81.5	3948	1.74	0.12
53	Stigmasteryl tosylate	63.6	3984	1.79	0.10
54	dl-α-Tocopherol/VE	84.3	4036	1.77	1.57
55	Campesterol	86.2	4148	2.075	1.19
56	Stigmasterol	84.7	4188	2.17	3.35
57	γ-Sitosterol	84.7	4264	2.3	0.65
58	Cholest-4-en-3-one, 26-(acetyloxy)-	79.6	4316	2.545	0.52
59	4,22-Stigmastadiene-3-one	88.3	4352	2.6	0.66
60	Lupeol	84.7	4360	2.77	0.10
61	Vitamin E	78.2	4384	2.515	0.11
62	Cholest-4-en-26-oic acid, 3-oxo-	74.7	4408	2.775	0.35
63	Stigmastane-3,6-dione, (5α)-	78.2	4580	2.73	0.22
A1	1-Hydroxy-4-methylanthraquinone	820	3036	2.395	0.09
A2	9,10-Anthracenedione, 2-hydroxy-1-methoxy-	838	3208	2.736	0.05
A3	Isopeucenin	665	3248	2.435	0.07
A4	10-Hydroxy-5-methoxy-2-methyl-1,4-anthracenedione	647	3336	2.675	0.45
A5	1-Hydroxy-4-methylanthraquinone	796	3408	2.92	1.92
A6	Anthraquinone, 2,3,6,7-tetramethyl-	661	3800	2.755	0.05

Note: 1) Identified compounds are labeled in Fig. 2, Fig. 3D; 2) These compounds were identified through library matching without validation. The No. was determined based on the elution time. The same labels are used in Table 2, Table 3.

**Table 2:** Compounds tentatively identified in the solid-phase extraction (SPE) Elution fraction.

No.	Name	Similarity %	t_1D_ (s)	t_2D_ (s)	Area%
1	Hexanal	92.9	516	1.005	1.53
2	1-Penten-1-one, 2-methyl-	82.6	552	1.16	0.12
3	3-Furaldehyde	93.7	576	1.415	0.54
4	o-Xylene	92.3	644	1.06	1.02
5	2(3H)-Furanone, 5-methyl-	89.4	644	1.465	0.15
6	Cyclopentene, 1-ethenyl-3-methylene-	91	700	1.105	0.15
7	Oxime-, methoxy-phenyl-_	82.6	724	1.09	1.34
8	2-Cyclopenten-1-one, 2-hydroxy-	90.3	780	1.515	0.18
9	Benzaldehyde	91.6	840	1.545	0.37
10	Hexanoic acid	94.4	872	1.27	4.28
11	Phenol	96.1	888	1.425	0.31
12	Furan, 2-pentyl-	93.6	904	1.1	0.53
13	2-Propen-1-ol	93.2	912	2.085	0.54
14	Cyclohexene, 1-methyl-5-(1-methylethenyl)-	88.9	992	1.08	0.15
15	Benzyl Alcohol	90.8	1008	1.56	2.00
16	2,4,6-Cycloheptatrien-1-one, 4-methyl-	90.4	1020	1.62	0.22
17	Acetophenone	86.6	1076	1.625	0.11
18	Heptanoic acid	93.3	1080	1.24	0.33
19	Benzocyclobuten-1(2H)-one	88.7	1092	1.595	0.70
20	Phenylethyl Alcohol	91.3	1120	1.57	0.18
21	4-Nonenal, (E)-	87.1	1136	1.235	0.61
22	Nonanal	92.1	1152	1.22	0.17
23	(Z,Z)-3,6-Nonadienal	85.8	1152	1.33	0.11
24	Levoglucosenone	88.4	1180	2.02	2.23
25	2,6,6-Trimethyl-2-cyclohexene-1,4-dione	89.9	1244	1.64	0.25
26	2,3,5-Trioxabicyclo[2.1.0]pentane, 1,4-bis(phenylmethyl)-	84.6	1268	1.75	0.33
27	Benzoic acid	95	1284	1.7	1.24
28	Octanoic Acid	94.1	1296	1.275	0.92
29	Borneol	92.1	1300	1.335	1.49
30	Ethanone, 1-(3-methylphenyl)-	94.4	1336	1.63	0.16
31	α-Terpineol	91.6	1348	1.325	0.16
32	1,4:3,6-Dianhydro-α-d-glucopyranose	92.8	1384	2.065	0.33
33	Benzofuran, 2,3-dihydro-	83.8	1400	1.67	15.38
34	Benzothiazole	93	1420	1.915	0.13
35	Cyclohexane, isothiocyanato-	86.4	1440	1.59	0.12
36	Ethosuximide	82.4	1472	1.855	0.11
37	2,6-Octadien-1-ol, 3,7-dimethyl-, (E)-	88.8	1476	1.32	0.11
38	Nonanoic acid	92.2	1496	1.32	0.71
39	Bicyclo[3.3.1]nonan-2-one, 1-methyl-9-(1-methylethylidene)-	81.2	1540	1.27	0.11
40	Indole	89	1564	1.975	0.10
41	2-Methoxy-4-vinylphenol	96.7	1600	1.69	5.74
42	Hydroxymethyl 2-hydroxy-2-methylpropionate	86.3	1616	0.685	0.58
43	Benzaldehyde, 4-hydroxy-	96.7	1692	2	0.18
44	5-Oxotetrahydrofuran-2-carboxylic acid	89.2	1696	1.7	0.31
45	Benzoic acid, 4-formyl-, methyl ester	93.2	1704	1.91	1.61
46	Vanillin	95.6	1768	2.06	0.81
47	4-(1,2-Dimethyl-cyclopent-2-enyl)-butan-2-one	83.9	1848	1.78	0.15
48	Phenol, 2-methoxy-4-(1-propenyl)-	94.5	1864	1.675	0.13
49	Ethanone, 1-(4-hydroxy-3-methoxyphenyl)-	93.1	1932	2.035	0.13
50	Phenol, 2,4-bis(1,1-dimethylethyl)-	92	1972	1.39	5.09
51	β-D-Glucopyranose, 1,6-anhydro-	93.3	2016	2.22	9.61
52	2(4H)-Benzofuranone, 5,6,7,7a-tetrahydro-4,4,7a-trimethyl-	87.8	2020	1.985	0.40
53	Fumaric acid, 4-chlorophenyl ethyl ester	86.3	2084	1.76	0.52
54	Vanilic acid hydrazide	82.6	2116	1.985	0.31
55	Cyclopenta[c]pyran-4-carboxylic acid, 7-methyl-, methyl ester	82.5	2124	1.87	0.29
56	3-Hydroxy-β-damascone	83.5	2164	1.74	0.18
57	-9-hydroxy-4,7-megastigmadien-3-one	89.1	2216	1.745	0.20
58	3-Oxo-α-ionone	81.9	2232	1.85	0.16
59	Benzaldehyde, 4-hydroxy-3,5-dimethoxy-	90.7	2240	2.29	0.49
60	3-Buten-2-one, 4-(4-hydroxy-2,2,6-trimethyl-7-oxabicyclo[4.1.0]hept-1-yl)-	90.9	2284	1.8	0.19
61	2-Propenoic acid, 3-(4-methoxyphenyl)-	90.4	2316	2.06	0.20
62	4-((1E)-3-Hydroxy-1-propenyl)-2-methoxyphenol	91.5	2372	2.15	1.45
63	2-Cyclohexen-1-one, 4-hydroxy-3,5,6-trimethyl-4-(3-oxo-1-butenyl)-	88.9	2452	2.055	0.38
64	1,2-Benzenedicarboxylic acid, butyl 2-methylpropyl ester	84.8	2560	1.675	0.14
65	n-Hexadecanoic acid	88	2692	1.345	0.73
66	7-Nonenamide	83.4	2696	1.69	0.15
67	Dodecanamide	91.6	2712	1.605	0.69
68	9,10-Anthracenedione, 2-methyl-	90.7	2912	2.405	0.14
69	1,E−11,Z-13-Octadecatriene	88	2932	1.435	0.53
70	9-Octadecenamide, (Z)-	86.4	3220	1.72	1.91
71	2-(Hydroxymethyl)anthraquinone	80.3	3340	2.985	0.28

Note: 1) The identified compounds are labeled in Fig. 2, Fig. 3B.

**Table 3:** Compounds tentatively identified in the solid-phase extraction (SPE) Unretained fraction.

No.	Name	Similarity %	t_1D_ (s)	t_2D_ (s)	Area %
1	Bis(2-chloroethyl) ether	93.4	1044	1.215	0.45
2	Propanoic acid, 2-oxy-	95.9	1164	0.93	1.20
3	Acetic acid, -oxy-	94.6	1188	0.955	0.88
4	Propanoic acid, 2-oxo-3-	95.4	1208	0.94	0.25
5	Acetic acid	86.6	1316	0.95	0.27
6	1-Undecanol	91.9	1388	0.96	0.10
7	1,2-Ethandimine, N,N′-ditrifluoroacetyl-	90.9	1396	1.49	0.29
9	2-Methoxyphenol	92.1	1456	1.235	0.14
10	Benzoic acid	88.3	1492	1.28	2.28
11	Pentadecane	90.1	1552	0.925	0.18
12	Glycerol	87	1560	0.915	0.99
13	Butanedioic acid	91	1620	1.115	0.45
14	Propanoic acid	87.3	1668	0.975	0.90
15	2-Butenedioic acid (E)-	92.6	1680	1.06	0.25
16	4-Fluoro-3-trifluoromethylbenzoic acid, anhydride	86.1	1712	1.06	0.21
17	Benzeneacetic acid	88	1932	1.21	0.33
18	Malic acid	87.7	1956	1.07	0.36
19	Proline	92	2008	1.355	0.20
20	Threonic acid	84.4	2064	0.955	0.36
21	Hexadecane	91.3	2292	1.005	0.52
22	Heptadecane, 2,6,10,15-tetramethyl-	88.4	2304	0.99	0.36
23	Eicosane	80.5	2452	1.03	0.12
24	Azelaic acid	85.2	2456	1.19	0.12
25	Isopropyl Myristate	91.5	2488	1.2	0.63
26	Benzoic acid	83.9	2504	1.175	0.14
27	Tetradecanoic acid	93.2	2528	1.125	0.49
28	α-D-Galactofuranose, 1,2,3,5,6-pentakis-	83.1	2568	0.955	2.91
29	Nonadecane, 2-methyl-	90.4	2628	1.025	0.86
30	Mannonic acid, 2,3,5,6-tetrakis-O-lactone	87.9	2640	1.075	0.41
31	Lyxose	86.2	2652	1.05	0.11
32	Cinnamic acid	90.6	2672	1.34	0.95
33	d-Galactose, 2,3,4,5,6-pentakis-O-methyloxyme, (1Z)-	88.1	2700	0.9	0.60
34	Glucopyranose, pentakis-O	92	2768	0.975	2.04
35	Ribonic acid, 2,3,4,5-tetrakis-O	83.3	2804	0.95	2.23
36	Hexadecanoic acid	89.1	2812	1.195	2.76
37	Oleanitrile	91.1	2860	1.45	0.15
38	9,12-Octadecadienoic acid (Z,Z)-	88.9	3032	1.24	0.24
39	Octadecanoic acid	86.8	3072	1.19	0.29
40	9-Octadecenamide	86.9	3216	1.645	0.24
41	Hexanedioic acid	91.6	3260	1.36	1.36
42	Eicosanoic acid	84.7	3308	1.14	0.15
43	1,2-Benzenedicarboxylic acid, mono(2-ethylhexyl) ester	81.1	3436	1.645	1.23

Note: 1) Identified compounds are labeled in Fig. 2, Fig. 3C.

**Fig. 2:**
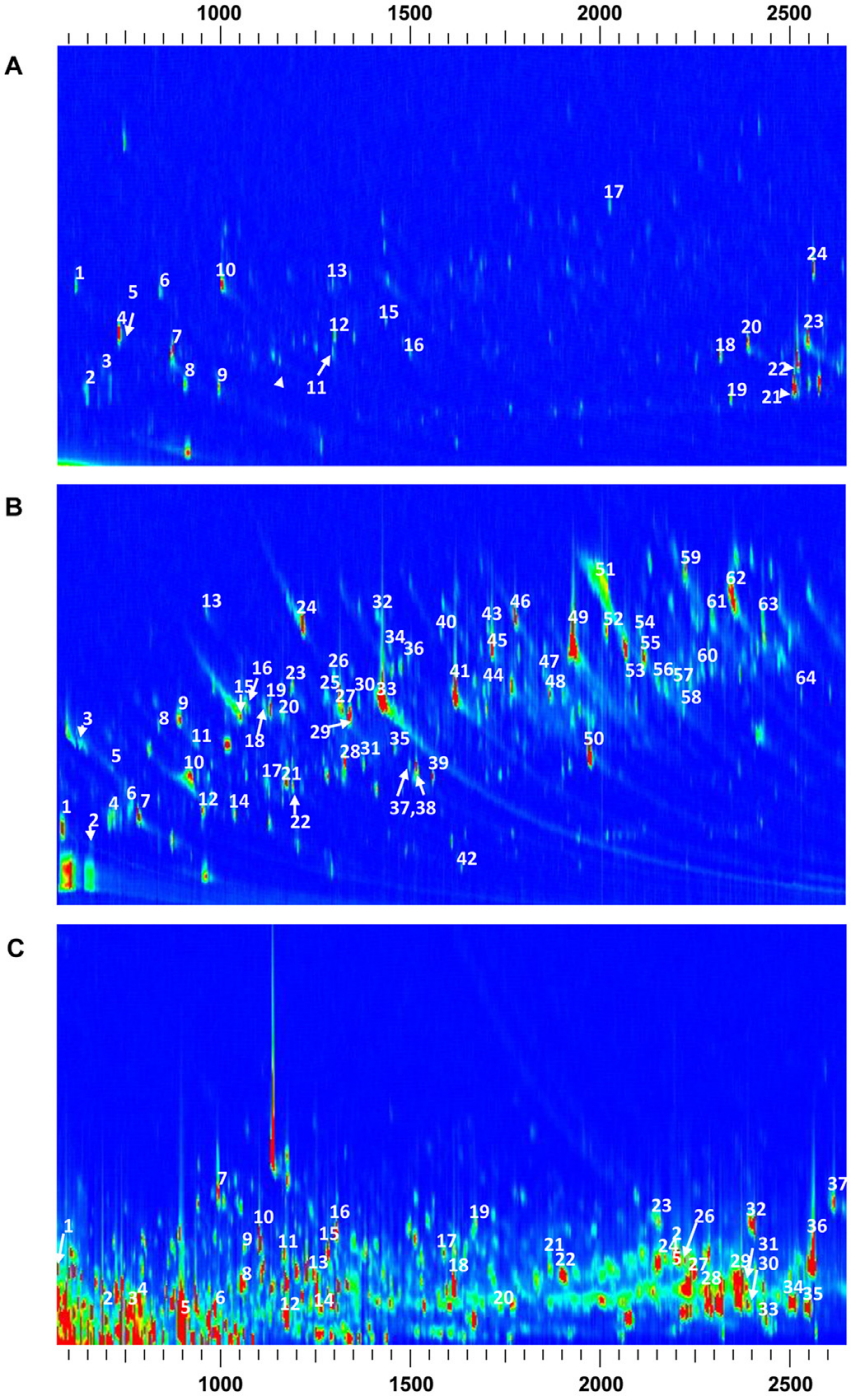
Two-dimensional gas chromatography-time-of-flight mass spectrometry (GC ​× ​GC-TOF/MS) analysis of *Hedyotis diffusa* in the time range of 500–2500 ​s ​A. Dichloromethane (DCM) extract; B. Solid-phase extraction (SPE) Elution fraction; C. SPE unretained fraction. Note: The No. Of the spots are consistent with the serial numbers in the corresponding table.

**Fig. 3:**
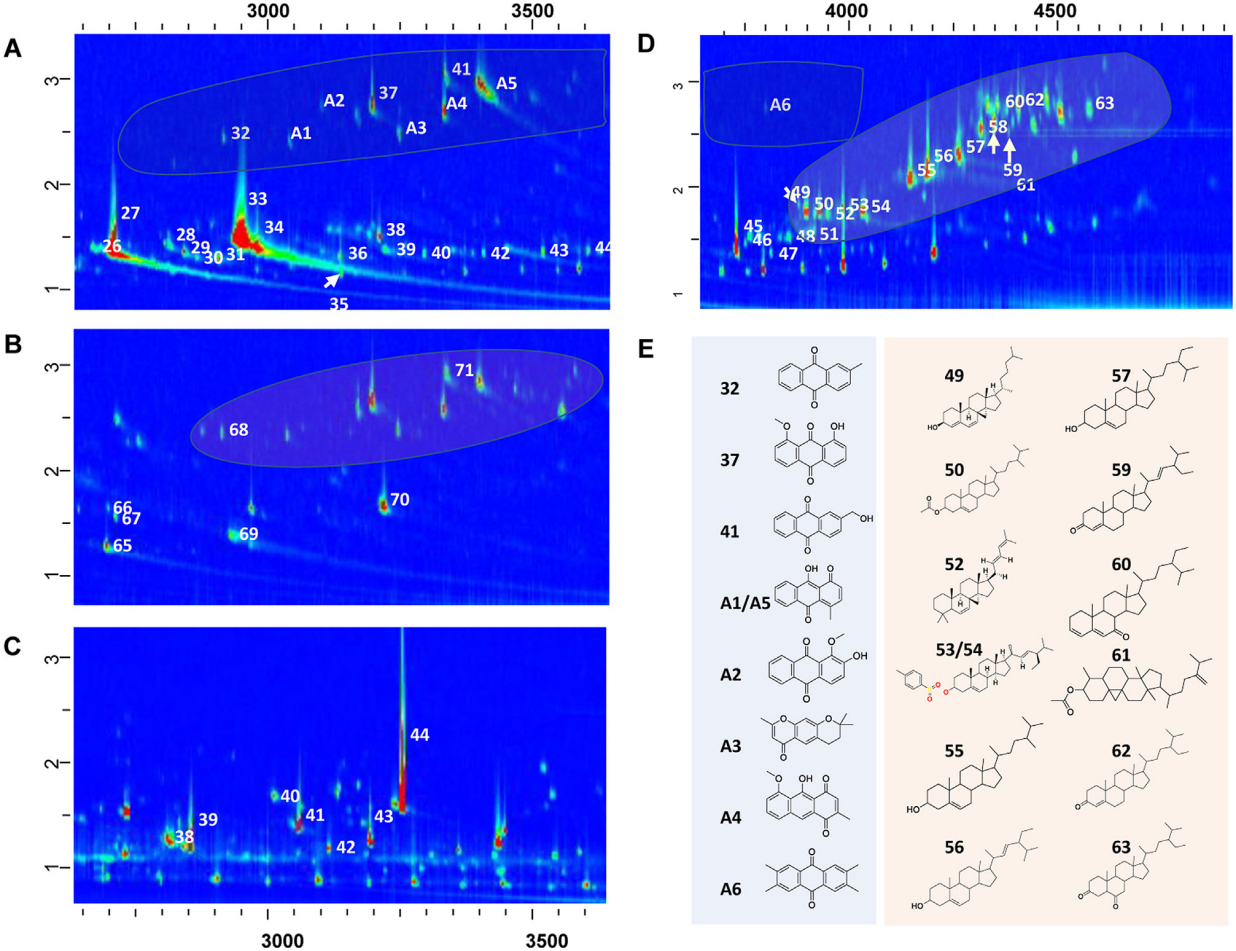
Identification of chemicals in the time range of 2600–3700 ​s ​A. Dichloromethane (DCM) extract; B. Solid phase extraction (SPE) Elution fraction; C. SPE Unretained fraction. D. DCM extract in the range of 3700–4800 ​s. E. Tentatively identified anthraquinones and terpenoids. Note: The No. Of the spots are consistent with the serial numbers in the corresponding table.

To decrease the laborious compound identification process, the number of peaks was controlled to less than 500 based on the signal-to-noise ratios (S/N) during peak integration. The numbers of peaks detected from the SPE Elution were 452 and 325 ​at ​S/N values ​> ​80 and ​> ​200, respectively. Library search results revealed 286 and 178 matching compounds, with a similarity of >80%. To decrease the number of false-positive results, an S/N of 200 was used for SPE elution and DCM extraction, and an S/N of 400 was used for SPE Unretained. The numbers of library-matched compounds were 185 and 483 for the DCM Extract and SPE Unretained, respectively, with a similarity >80%. The library-matched compounds accounted for 90.4%, 77.9%, and 43.7% of the total peak areas of the DCM extract, SPE elution, and SPE Unretained samples, respectively. Identified peaks with a similarity >80% and compounds with a peak area (MS detection) ​> ​0.1% are labeled in Fig. 2, Fig. 3. The compound information is presented in Table 1, Table 2, Table 3.

*Hedyotis diffusa* is rich in fatty acids. In total, 32 fatty acids and their esters were tentatively identified. These compounds accounted for 50.25% of the peak area in the DCM Extract. n-Hexadecanoic acid (27) and 9-octadecynoic acid (33) were the most abundant fatty acids, accounting for 19.88% and 19.50%, respectively. 2,3-Dihydro-benzofuran was the most abundant compound in the SPE Elution fraction, accounting for 15.38% of the peak area. Hexadecanoic and octadecanoic acids were found in the SPE Unretained, accounting for 2.76% and 2.71%, respectively. Wong et al. [3] used hydrodistillation extraction and GC/MS to identify 34 compounds, among which p-vinylphenol (22.2%), p-vinylguaiacol (18.6%), and linalool (13.6%) were the most abundant. Jian et al. [4] identified 43 components, of which the major component was hexadecanoic acid (48.89%), followed by pentadecanoic acid (6.11%) and d-limonene (5.74%). Liu et al. [5] and Wang et al. [6] identified 33 compounds using LC-QTOF/MS. Want and Chen [26,27] used ultra-HPLC-QTOF/MS to identify 104 chemical compounds. We developed 2D-LC-QTOF/MS methods that can separate more than 200 compounds [11,28]. Unfortunately, a well-established MS library is needed to identify these compounds.

A limitation of the present study is that the identified compounds were not validated using the linear retention index (LRI). The LRI value is a useful first measure of molecular identity. However, LRIs should be applied in a rigorous manner, and both the first and second columns in the GC ​× ​GC experiment must be measured [29]. Moreover, compounds from the SPE Unretained fraction were identified as their -trimethylsilyl form, making identification difficult.

### 3.4 Group-type pattern of separation

Iridoid glycosides are responsible for the pharmacological activities of *H. diffusa* [30]. High concentrations of fatty acids (n-hexadecanoic acid and 9-octadecynoic acid), anthraquinones, and terpenoids may also contribute to its biological functions [21,22]. To study their structure or biological activity, these compounds must be isolated; for example, Hung et al. [31] studied 5.2 ​kg ​*H. diffusa* whole plants and isolated seven new anthraquinones. Chemical profiling of *H. diffusa* provided preliminary information regarding its compounds. Group type separation patterns were observed in the contour plot of the DCM Extract. The steroid and anthraquinone zones are marked with shadows in Fig. 3. Anthraquinones with similarity <80% or content <0.1% are marked with the initial letter A in Table 1 and Fig. 3A. Nine anthraquinones were identified. Twenty-four anthraquinones and four sterols have been reported in *H. diffusa* [32]. Through GC ​× ​GC-TOF/MS analysis of the DCM Extract, 12 sterols were tentatively identified, which is three-fold more than the reported number [3].

Identification of the anthraquinone and steroid zones via GC ​× ​GC-TOF/MS analysis yielded interesting results. The group-type pattern in 2D chromatography are useful for structural identification. The zones of anthraquinone and steroids suggest the presence of various anthraquinone compounds that have not been reported. Therefore, it is important to isolate, structurally examine, and evaluate compounds after the present chemical profiling.

A limitation of the present study is that GC-MS only analyzed volatile compounds. Information on nonvolatile compounds remains lacking. Aqueous extracts may contain nonvolatile compounds that can contaminate the liner of a GC system. Attention should be given to the sample preparation technique.

The aim of the present study was to investigate the feasibility of combined sample preparation coupled with GC ​× ​GC separation. The GC ​× ​GC conditions could be optimized for each sample preparation technique to achieve better separation and obtain better library-matching results in the following investigations. Furthermore, studies on qualitative analysis using LRIs or quantitative analysis using external or internal standards require additional in-depth investigation.

## 4 Conclusion

We developed a combined sample preparation technique coupled with the high-resolution capabilities of GC ​× ​GC-TOF/MS to explore the chemical profile of *H. diffusa.* DCM and aqueous extraction tandem SPE fractionation yielded nonpolar, moderately polar, and polar compounds. Fractionation of the sample decreased its complexity and the degree of coelution in the GC ​× ​GC analysis to some extent. The results of GC ​× ​GC analysis constituted a comprehensive chemical profile of *H. diffusa.* Moreover, the group-type patterns observed in the GC ​× ​GC contour plots, particularly for steroids and anthraquinones, facilitated their identification. Therefore, our method is useful for chemical profiling of natural products.

## Ethics approval

Not applicable.

## Fundings

Not applicable.

## CRediT authorship contribution statement

**Duxin Li:** Writing – original draft, Investigation, Data curation, Conceptualization. **Xinying Du:** Writing – original draft, Data curation. **Wanru Bai:** Writing – original draft, Data curation. **Oliver J. Schmitz:** Writing – review & editing, Project administration, Methodology, Conceptualization.

## Data availability

The data used in this study are available upon request from the corresponding author upon reasonable request.

## Declaration of competing interest

The authors declare that they have no known competing financial interests or personal relationships that could have appeared to influence the work reported in this paper.
